# A Primary Dead-Weight Tester for Pressures (0.05–1.0) MPa

**DOI:** 10.6028/jres.108.013

**Published:** 2003-04-01

**Authors:** Kamlesh Jain, Walt Bowers, James W. Schmidt

**Affiliations:** National Physical Laboratory, New Delhi, India; National Institute of Standards and Technology, Gaithersburg, MD 20899-8360

**Keywords:** dead-weight tester, piston/cylinder assembly, piston gage, pressure measurement, primary pressure standards

## Abstract

Recent advances in technology on two fronts, 1) the fabrication of large-diameter pistons and cylinders with good geometry, and 2) the ability to measure the dimensions of these components with high accuracy, have allowed dead-weight testers at the National Institute of Standards and Technology (NIST) to generate pressures that approach total relative uncertainties previously obtained only by manometers. This paper describes a 35 mm diameter piston/cylinder assembly (known within NIST as PG-39) that serves as a pressure standard in which both the piston and the cylinder have been accurately dimensioned by Physikalisch Technische Bundesanstalt (PTB). Both artifacts (piston and cylinder) appeared to be round within ±30 nm and straight within ±100 nm over a substantial fraction of their heights. Based on the diameters at 20 °C provided by PTB (±15 nm) and on the good geometry of the artifact, the relative uncertainties for the effective area were estimated to be about 2.2 × 10^−6^ (1σ).

## 1. Introduction

The pressure standard in the atmospheric pressure range at the National Institute of Standards and Technology (NIST) is presently established using mercury manometers [1–4]. However, recent developments in the fabrication of large-diameter high-quality piston/cylinder assemblies and recent advances in dimensional metrology have allowed the pressure measurement community to contemplate primary pressure standards that are based on dimensional measurements of pistons and cylinders whose uncertainties in generated pressures could approach the best manometers.

The Pressure and Vacuum Group at NIST has recently acquired new dimensional measurements of high quality from Physikalisch Technische Bundesanstalt (PTB) [5,6] that were taken from a piston gage with a history going back about 12 years [7,8]. The new measurements have yielded substantially reduced uncertainties for the effective area compared with the previous determinations. This gage has a relatively large diameter (≈35 mm), which means that PTB’s stated uncertainty on length measurements (±15 nm) would allow the diameter of each piece to be determined with a relative standard uncertainty less than 0.5 × 10^−6^, (1σ). This would translate to a relative standard uncertainty in area of 1.0 × 10^−6^, (1σ).

Dimensional measurements allow a direct determination of the effective area of this gage without referring to another pressure standard for its calibration. For smaller diameter gages the diameter of the cylinder is typically determined by a cumbersome procedure invented by Johnson and Newhall [9] which is described by Heydemann and Welch [10] and is referred to as a controlled clearance technique. Other equally important aspects for the translation of these very accurate linear dimensions to an accurate effective area are that both pieces constituting the present gage possessed excellent geometry and there was a relatively small clearance between piston and cylinder. These three conditions, 1) accurate dimensional measurement capability from the comparator at PTB, 2) good geometry of the artifact and 3) small clearance allows the effective area when used as a pressure generator to be determined with a relative standard uncertainty u(A)/A ≈ ±1.4 × 10^−6^, (1σ).

A value for the effective area distilled from all the information in this report agrees with a recent value obtained via NIST’s Ultrasonic Interferometer Manometer (UIM) [11] within 2.5 × 10^−6^ and it agrees within 1 × 10^−6^ of dimensional measurements performed at NIST some years ago [8].

Because NIST’s Pressure and Vacuum Group uses a reference temperature of 23 °C whereas the dimensional measurements were done at 20 °C it was necessary to obtain an accurate value for the thermal expansion in order not to degrade the accuracy when operating the gage at 23 °C. A special oven/cooler was constructed to measure the thermal expansion.

## 2. Apparatus

**Fig 1:**
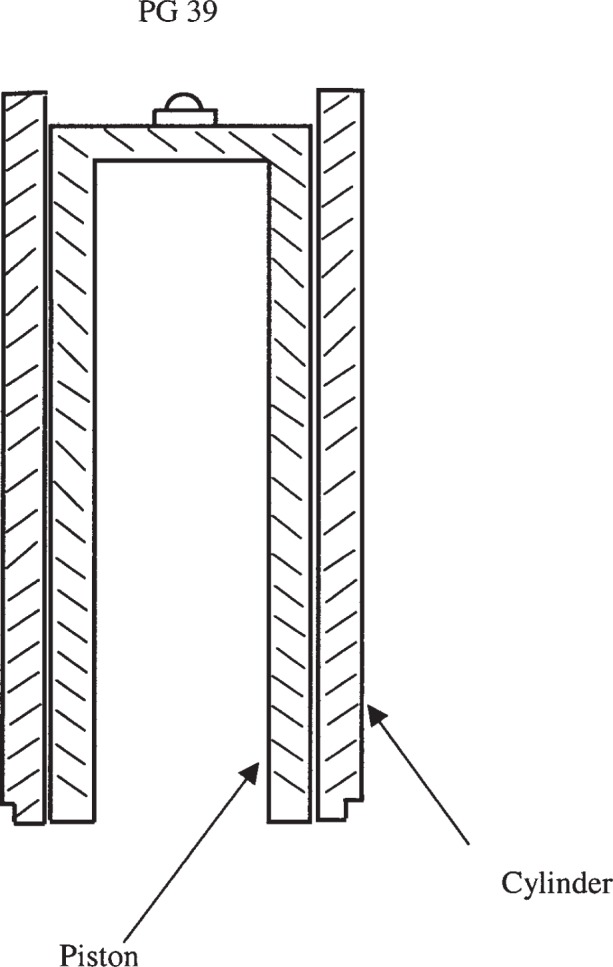
Schematic representation of the 35 mm piston/cylinder assembly. Both piston and cylinder are made from single castings of tungsten carbide.

For the present measurements we used a piston and a close fitting cylinder with large (35 mm) diameters made by the Ruska Instrument Corporation^1^. (See Fig. 1.) Known within NIST as PG-39, both piston and cylinder were made of tungsten carbide. When used as a pressure generator the assembly employs a conventional design with the usual floating piston. An important feature of the gage is that both piston and cylinder are fashioned from single blocks of tungsten carbide rather than relying on a bimetallic construction. With careful handling we expect this feature to provide good stability over extended periods.

For the dimensional measurements we relied on the relatively new state of the art comparator at PTB, Braunschweig Germany, which has the capability of measuring both diameter and straightness of cylinders using a probe contact technique with high accuracy. Diameters via this comparator were obtained on both piston and cylinder [6]. Roundness measurements were obtained using other equipment at PTB.

Other specialized apparatus was used for auxiliary measurements: i) an oven/cooler for measurements of the thermal expansion coefficient, ii) capacitance measurements between the piston and cylinder for estimates of the crevice width, and iii) ultrasound for measurements of Young’s modulus of the piston and cylinder.

Rather than attempt to determine the linear expansion coefficient of the tungsten carbide material for the individual components with laser interferometry for example, it was easier to use our expertise in pressure metrology and determine the areal expansion coefficient through a direct comparison of pressure with a reference piston gauge. A temperature controlled environmental chamber (oven/cooler) was constructed for the 35 mm piston/cylinder assembly and base and was used to accurately measure the thermal expansion coefficient of the piston/cylinder assembly by placing PG-39 inside the chamber and using another piston gage outside the chamber as a reference. The chamber was capable of better than ±0.005 K stability. The temperature of the chamber could be controlled between 10 °C and 40 °C using a Peltier element and could be measured with a calibrated thermometer to better than ±0.02 K. With the piston/cylinder assembly inside, however, the chamber was operated only between 15 °C and 40 °C in order to avoid possible damage to the piston and cylinder. In general, a longer temperature span yields a more accurate expansion coefficient. Thermal gradients within the oven were estimated to be less than ±0.1 °C.

For crevice width measurements, a capacitance gauge with ±0.1 nF resolution was used to measure the capacitance between the piston and cylinder in its pressure column. One electrode was attached to the base of the assembly and electrically at the same ground potential as the cylinder. The other electrode was connected to the top of the piston through a small cup that contained a tiny amount of mercury in order to minimize extraneous non-axial forces on the cylinder assembly. The capacitance method is currently under investigation within the Pressure and Vacuum Group as a means of measuring the clearance in other gages.

For estimating Young’s modulus, *E*, the speed of sound in the tungsten carbide piston was measured using an ultrasonic pulse launched at one end of the piston. From its reflection at the other end and subsequent return, the pulse was detected and the total time of flight was measured from which the speed of sound was determined. Young’s modulus was obtained from the speed of sound, *c*, and the density *ρ* [12]:

$$E=\rho c^{2}.$$ (1)

Similar measurements were made on the cylinder.

## 3. Characterization From Dimensional Measurements

**Fig. 2:**
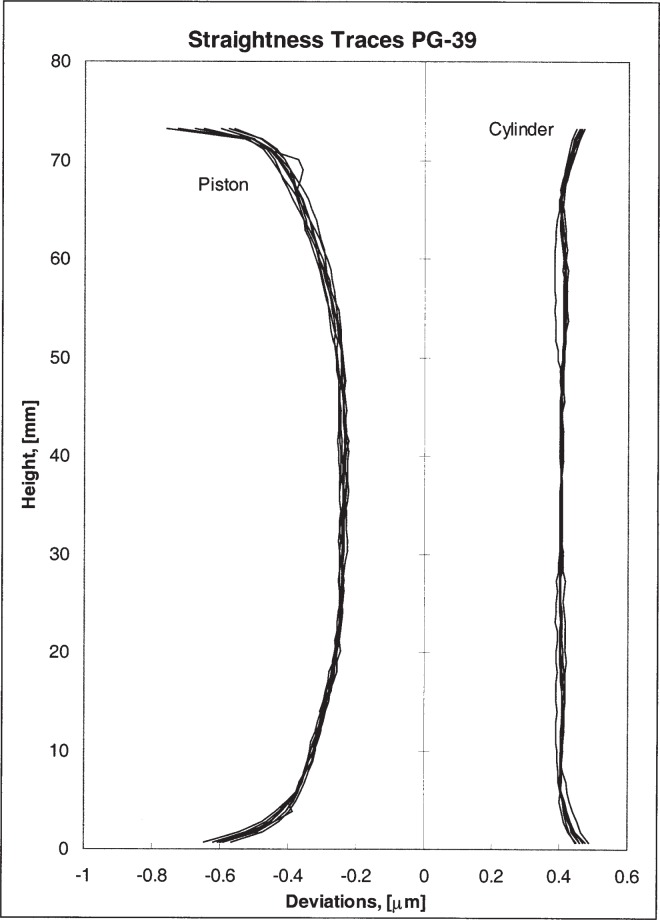
Straightness Traces of PG-39. Deviations from straight lines were measured at 0°, 45°, 90°, 135°, 180°, 225°, 270°, 315°, and 360°. Straightness data were coupled with absolute diameters and the roundness data to construct the traces in this figure, which are referenced to an absolute scale.

**Fig. 3:**
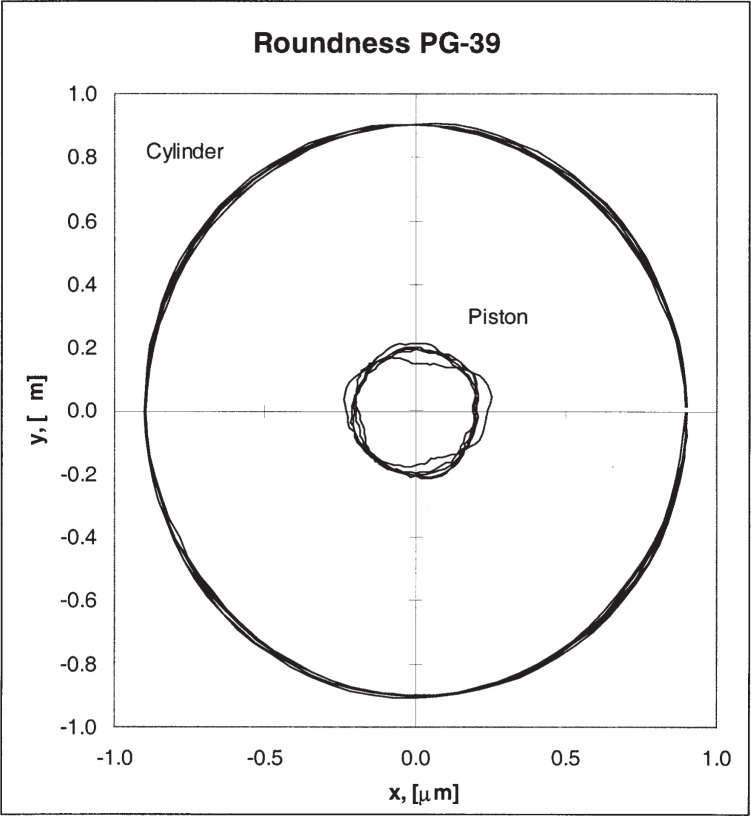
Roundness Traces of PG-39. Deviations from circles were measured at elevations (3.0, 18.75, 37.4, 56.25, and 72.0) mm from the bottom. Roundness data were coupled with absolute diameters and the straightness data to construct the traces in this figure, which are referenced to an absolute scale.

The PTB measured the piston and cylinder using their relatively new state-of the art comparator [5]. Diameters were measured along two directrices (two longitudes, 0° to 180° and 90° to 270°) for both pieces. Diameters were obtained at two places in both vertical planes, or four diameters on the piston and four diameters on the cylinder. All diameters were measured near 20 °C and adjusted to the reference temperature of 20 °C. A full set of straightness data was obtained from both piston and cylinder using the comparator. (See Fig. 2.) Roundness data were obtained using a separate device. (See Fig. 3.)

### 3.1 Direct Averages

We averaged the diameters supplied by PTB for both piston and cylinder, and this yielded values for the areas of each component at the reference temperature 20 °C:

$$A_{0p,20}=\pi D_{p}^{2}/4\approx \pi {\left(35.822\;875\pm 0.000\;032\right)}^{2}\;{\mathrm{mm}}^{2}/4,$$ (2a)

and

$$A_{0c,20}=\pi D_{c}^{2}/4\approx \pi {\left(35.824\;318\pm 0.000\;017\right)}^{2}\;{\mathrm{mm}}^{2}/4,$$ (2b)

Here *D*_p_ and *D*_c_ are the average diameters of the piston and cylinder, respectively. The ambient pressure (1 atmosphere) effective area of the assembly derived from these measurements at 20 °C is:

$$A_{0,20}=\left(A_{0p,20}+A_{0c,20}\right)/2=\left(1007.9251\pm 0.0012\right)\;{\mathrm{mm}}^{2}.$$ (3)

The uncertainty listed represents a relative uncertainty of 1.2 × 10^−6^ (1σ) at ambient pressure and is obtained from the type B uncertainty from the dimensional measurements root sum squared with the variance of the mean of the diameters. (See Tables 1–3.) The type B uncertainties were added together algebraically because these could be correlated. This area compares very favorably with the area obtained from dimensions measured by the NIST Precision Engineering Division in 1989, (1007.926 ± 0.011) mm^2^, @ 20 °C [7,8].

### 3.2 Numerically Integrated Results

All of the information, absolute diameters at four places, roundness traces at five heights, and straightness traces at eight angles was put together in the form of what is sometimes called a “birdcage” that represented the piston and another set of information to represent the cylinder. Cylindrical harmonics were then fit to the data in order to obtain analytic functions *r*_p_(*z*,*θ*) and *r*_c_(*z*,*θ*) for the surfaces where *z* is the vertical coordinate and *θ* is the azimuth angle. Using *r*_p_(*z*,*θ*) and *r*_c_(*z*,*θ*), a numerical integration of forces acting over the surface of the piston was performed with Dadson et al.’s work serving as a guide [13]. These authors divide the forces into three categories, 1) a basal force acting upward on the base of the piston, 2) a vertical component of the normal forces acting on the sides of the piston if it is other than perfectly straight and vertical, and 3) a force from viscous gas flowing upward and exerting a vertical drag on the piston.

#### 3.2.1 The Piston Base, *A*_base_

The base area of the piston, *A*_base_, was obtained by a numerical integration of the analytical function *r*_p_(*z*,*θ*):

$$A_{\text{base}}=\frac{1}{2}\int_{0}^{2\pi }r_{p}^{2}\left(0,\theta \right)d\theta =1007.865\;{\mathrm{mm}}^{2},$$ (4)

where *r*_p_(*z* = 0,*θ*) is the piston radius at the base of the piston.

#### 3.2.2 Shape Contribution δ*A*_s_

The change in *r*_p_(*z*,*θ*) with respect to height introduces an additional vertical force given by the following equation:

$$\begin{array}{c} \delta F_{s}=P_{0}\int_{0}^{2\pi }\left[r_{p}^{2}\left(0,\theta \right)-r_{p}^{2}\left(L,\theta \right)\right]d\theta /2\\ +\int_{0}^{L}\int_{0}^{2\pi }P\left(z\right)\frac{dr_{p}}{dz}r_{p}\left(z,\theta \right)d\theta dz. \end{array}$$ (5)

Here *P*_0_ is the pressure at the top, *P*_1_ is the pressure at the bottom of the piston, and *P*(*z*) is the pressure as a function of height within the crevice and *L* is the length of the crevice. The contribution to the effective area from the shape of the sides of the piston is then:

$$\delta A_{s}=\delta F_{s}/\left(P_{1}-P_{0}\right).$$ (6)

Numerically integrating the derivative of the fitting function, d*r*_p_/d*z*, as indicated above using a pressure profile, *P*(*z*), derived from the Poiseuille flow equation gives an increase in the effective area:

$$\delta A_{s}\sim +0.0167\;{\mathrm{mm}}^{2},$$ (7)

with respect to the area at the base of the cylinder. The pressure profile was derived assuming an average crevice width at each height

$$\bar{h}\left(z\right)=\frac{1}{2\pi }\int_{0}^{2\pi }h\left(z,\theta \right)d\theta ,$$ (8)

where the crevice width is *h*(*z*,*θ*) = *r*_c_(*z*,*θ*) − *r*_p_(*z*,*θ*). In Eq. (5) a gas density linear in pressure was also assumed. In this case:

$$P\left(z\right)=\sqrt{P_{1}^{2}-\frac{P_{1}^{2}-P_{0}^{2}}{I_{z}}\int_{0}^{z}\frac{dz\prime }{\bar{h}{\left(z\prime \right)}^{3}}},$$ (9)

where *P*_1_ and *P*_0_ are the pressures at the bottom and the top of the crevice, respectively. The definite integral *I*_z_ is:

$$I_{z}=\int_{0}^{L}\frac{dz\prime }{\bar{h}{\left(z\prime \right)}^{3}}.$$ (10)

#### 3.2.3 The Flow Contribution *δA*_f_

The flow of gas up through the crevice between the piston and cylinder contributes a drag force that must be accounted. Assuming Poiseuille flow in the crevice the drag force is:

$$\delta F_{f}\approx -\frac{1}{2}\int_{0}^{2\pi }d\theta \int_{0}^{L}dzr_{p}\left(z,\theta \right)\frac{dP\left(z\right)}{dz}h\left(z,\theta \right).$$ (11)

Numerically integrating Eq. (11) using the fitting functions *r*_c_(*z*,*θ*) and *r*_p_(*z*,*θ*) with the same pressure profile as in the previous section and converting the results to fractional area gives:

$$\delta A_{f}=\delta F_{f}/\left(P_{1}-P_{0}\right)\approx +0.0449\;{\mathrm{mm}}^{2}.$$ (12)

The drag force (since it is acting up-ward in this case) will serve to increase the area of the piston by an amount of about 44.6 × 10^−6^.

Adding the contributions from Eqs. (4), (7) and (12) gives:

$$A=A_{\text{base}}+\delta A_{s}+\delta A_{f}=1007.9267\;{\mathrm{mm}}^{2}.$$ (13)

#### 3.2.4 Uncertainty in the Numerical Integration of *A*_base_, *δA*_s_, *δA*_f_

The principal uncertainty in the numerical calculation of *A*_base_, *δA*_s_, *δA*_f_ arises from the uncertainty in the dimensional measurements and the simplifying assumptions involved in calculating the pressure profile. A sensitivity check on the integration’s dependence on the input parameters showed that the uncertainty in the average radius of the piston, *u*(*r*_p_), produced about a 0.43 × 10^−6^ uncertainty in the area of the gauge. A similar check of the uncertainty of the derivative d*r*_p_/d*z* ≈ 0.4 nm, showed about a 0.19 × 10^−6^ contribution to the uncertainty in the effective area. Similar sensitivity checks on the radius of the cylinder, *r*_c_, and d*r*_c_/d*z*, produced 0.42 × 10^−6^ and 0.30 × 10^−6^ shifts in the effective area, respectively. With regard to the calculation of the pressure profile, the simplifying assumption of Eq. (8) was checked by assuming instead that:

h(z)=max[h(z,θ)], (14)

in Eq. (9), with the result that d*A*/*A* changed by about 0.1 × 10^−6^ mm^2^/mm^2^. Several integrations were done in which the cylinder was rotated with respect to the piston. This resulted in small differences, <0.15 × 10^−6^. Moving the piston and cylinder’s vertical position relative to one another by 3.5 mm, resulted in a 1.0 × 10^−6^ change in effective area. Root sum squaring the seven contributions to the uncertainty in the effective area, namely, *u*(*r*_c_), *u*(d*r*_c_/d*z*), *u*(*r*_p_), *u*(d*r*_p_/d*z*), *u*(*h*), *u*(*θ*_p_) and *u*(*z*_p_ − *z*_c_) adds an uncertainty of 1.2 × 10^−6^.

Lastly, with regard to the flow contribution, another model for the flow was assumed [14]. This model takes into account transition flow within the clearance and generally gives an effective area slightly smaller than the Poiseuille flow model. This alternative model resulted in an effective area 2.5 × 10^−6^ below the Poiseuille flow model. The average value of the effective area for the two models is:

$$A_{\mathrm{NI}}=\left(1007.925\;2+0.002\;2\right)\;{\mathrm{mm}}^{2}.$$ (15)

We have taken as an uncertainty for model dependent crevice effects, the standard deviation obtained from the two models which is 1.8 × 10^−6^. The uncertainty in Eq. (15) is obtained by combining the uncertainty of the numerical integration, 0.0012 mm^2^, with the flow-model uncertainty, 0.0018 mm^2^ in quadrature.

Note that the uncertainty given in Eq. (15) would result in an uncertainty in generated pressure of 2.2 × 10^−6^ *P*. This however, does not include uncertainties from mass loading and other “in use” effects when used in a secondary calibration.

## 4. Auxiliary Measurements

### 4.1 Thermal Expansion Coefficient

For operation of the gage at temperatures other than 20 °C a thermal expansion coefficient for the piston/cylinder assembly’s area is needed. With the special environmental chamber constructed to fit the gage, a coefficient was found to be:

$$\alpha =\left(8.754\pm 0.03\right)\times 10^{-6}/K,$$ (16)

where the uncertainty represents a coverage factor (*k* = 1). Thus when used near the Pressure and Vacuum Group’s reference temperature 23 °C an additional uncertainty of only (23 °C − 20 °C) × (0.03 × 10^−6^/K) = 0.09 × 10^−6^ is incurred.

### 4.2 Pressure Coefficient

For operation of the gage over the intended pressure range, (0.05 to 1.0) MPa, a pressure coefficient is needed. It can be estimated from elasticity theory using Young’s modulus and Poisson’s ratio [15] or obtained from calibrations to other gages. We obtained values for Young’s modulus from speed of sound measurements on the piston and cylinder [12,16]. The speed of sound was measured ultrasonically and found to be (6380 ± 140) m/s for the piston and (6580 ± 146) m/s for the cylinder (1σ). With a material density of 14 × 10^3^ kg/m^3^, Eq. (1) yields Young’s moduli of (5.70 ± 0.24) × 10^11^ Pa and (6.06 ± 0.26) × 10^11^ Pa for the piston and cylinder respectively, (1σ).

Jain et al. derived the pressure coefficients for both piston and cylinder for this gage using elasticity theory and the thick-wall formula [7]. (In that report the gage is referred to as NIST-9.) They used a value *b* = 8.0 × 10^−12^ Pa^−1^ for the pressure coefficient of the gage. No uncertainty was given but values from calibrations to other gages yield a spread of values between 2.8 × 10^−12^ Pa^−1^ and 5.18 × 10^−12^ Pa^−1^. An axi-symmetric finite element model produced a value (10 ± 2.0) × 10^−12^ Pa^−1^, based on a Young’s modulus of 6.0 × 10^11^ Pa and Poisson’s ratio 0.218. If one takes a square distribution of values for *b* between the lowest, 2.8 × 10^−12^ Pa^−1^, and highest values, 10 × 10^−12^ Pa^−1^, one obtains the value:

$$b=6.4\times 10^{-12}\;{\mathrm{Pa}}^{-1},$$ (17)

where the standard uncertainty is 2.1 × 10^−12^ Pa^−1^.

### 4.3 Clearance

The clearance, *h*, between the piston and cylinder can be determined using a variety of techniques and although they do not provide direct help in reducing the uncertainty of the effective area, based on the dimensional measurements, these other measurement techniques can provide consistency checks on the dimensional measurements. Primarily, the radial clearance can be obtained from the dimensions of the piston and cylinder, secondly via fall-rate measurements and thirdly via capacitance measurements.

#### 4.3.1 Via Dimensional Measurements

The dimensional measurements lead to an average clearance of:

$$h_{\text{Dim}}=\left(D_{c}-D_{p}\right)/2\sim \left(0.721\pm 0.016\right)\;µm,$$ (18)

where *h*_Dim_ is the clearance. The average diameters *D*_c_ and *D*_p_ were determined from direct dimensional measurements and were listed earlier.

#### 4.3.2 Via Fall-Rate Measurements

Fall-rate measurements, interpreted with the Poiseuille flow equation for a uniform crevice [17,18], were also used to obtain the clearance:

$$h_{\text{Poise}}={\left[12\frac{RP_{1}\eta L}{\left(P_{1}^{2}-P_{0}^{2}\right)}\times \frac{dz}{dt}\right]}^{1/3}.$$ (19)

Here *η* is the viscosity of the pressure fluid (nitrogen), *R* is the radius of the piston, *L* is the engagement length, *P*_0_ and *P*_1_ are the absolute pressures at the top and the bottom of the crevice respectively and d*z*/d*t* is the fall rate. This method has been used by Molinar and Vatasso [19], by Dolinskii et al. [20] and by Meyers and Jessup [21].

The fall-rates at several pressures are listed in Table 4. The clearance *h*_Poise_ from Eq. (19) is listed in the 4th column. These values for the clearance are seen to be about 30 % higher than the values obtained from dimensional measurement, *h*_Dim_, and from capacitance measurements, *h*_Cap_. (See below.) However, slip-flow phenomena have not been taken into account in Eq. (19). Slip flow has been used before in the interpretation of fall-rate data [22] and can be important in describing flow in narrow channels [23]. When slip flow is taken into account the apparent clearance is reduced by about 10 %:

$$h_{\text{Slip}}=\frac{h_{\text{Poise}}}{{\left(1+6K_{\text{Slip}}K_{n}\right)}^{1/3}},$$ (20)

where *K*_Slip_ is an accommodation coefficient taken to be 1.0 and *K*_n_ is the Knudsen number,

$$K_{n}=\frac{\lambda }{h},$$ (21)

and where λ is the mean free path,

$$\lambda =\frac{16}{5}{\left(\frac{R_{g}T}{2\pi M}\right)}^{1/2}\frac{\eta }{<P>}.$$ (22)

Here *R*_g_ is the gas constant, *T* is the thermodynamic temperature, *M* is the molar mass of the gas (N_2_), *η* is the viscosity of the gas and <*P*> is the average pressure in the crevice. When Eqs. (20) with Eq. (21) are used with *h*_Poise_ from Eq. (19), values for *h*_Slip_ result that are about (0.800 ± 0.110) μm. This is about 10 % larger than *h*_Dim_, but within the combined uncertainty of the different techniques. See Table 4.

#### 4.3.3 Via Capacitance Measurements

Lastly, clearances were determined using capacitance measurements [24]:

$$h_{\text{cap}}=\varepsilon_{0}K\frac{2\pi RL}{C\left(P\right)}.$$ (23)

Here *ε*_0_ is the permittivity of the vacuum, *K* is the dielectric coefficient of the pressure fluid (nitrogen), and *C* is the measured capacitance. For the interpretation of the capacitance measurements an ideal geometry was assumed, as was the case for the interpretations of the fall-rate measurements using the Poiseuille flow model. Minimal efforts were made to shield extraneous signals from the capacitance gauge. After transients had subsided, very stable operation was found with the piston only in the column and pressurized to a value near 4 kPa. The piston was allowed to float without spinning. Values for the capacitance ranged between 91 nF and 96 nF. Most of the time the piston seemed to self-center for long periods as indicated by the measured capacitance, which is at a relative minimum when the piston is centered. From time to time the values of capacitance would increase dramatically indicating that the piston was drifting off center. When more weights were added, some configurations were found to be stable, while others were unstable. The clearances obtained from the capacitance measurements were found to be:

$$h_{\text{cap}}\sim \left(0.725\pm 0.020\right)\;µm.$$ (24)

This is for a pressure of about 4 kPa generated by the piston only.

## 5. Summary

We have characterized a 35 mm dead-weight tester, known within NIST as PG-39, using dimensions obtained from PTB. An effective area was obtained by averaging the eight absolute diameters, four for the piston and four for the cylinder.

In addition a numerical integration of forces over the surface of the piston was performed and yielded a value about 1.6 × 10^−6^ higher than the simple average. For this integration, Poiseuille flow was assumed in the crevice. A second numerical integration was performed in which an alternative model for flow was assumed [14]. In this case the effective area was 0.9 × 10^−6^ lower than the simple average. Averaging the results of the two numerical integrations yields an effective area

$$A_{\mathrm{NI}}=\left(1007.925\;2\pm 0.002\;2\right)\;{\mathrm{mm}}^{2},$$ (25)

and is the recommended value @20 °C. The standard uncertainty given here also covers the averaged value obtained from the eight absolute diameters. For transferring this characterization to other gages, uncertainties from other sources will come into play and are not covered by this uncertainty.

For use at temperatures other than 20 °C, the thermal expansion coefficient for the effective area was measured in our laboratory in a controlled environmental chamber and was found to be *α* = (8.754 ± 0.03) × 10^−6^/K.

For use at higher pressures up to 1 MPa, a pressure coefficient was estimated using a variety of sources. The recommended value is

$$b=\left(6.4\pm 2.1\right)\times 10^{-12}\;{\mathrm{Pa}}^{-1}.$$ (26)

Auxiliary measurements (based on fall rates and capacitances) were made on the clearances between the piston and cylinder. These served as checks on the dimensional measurements. These measurements agreed with the dimensional measurement within their combined standard uncertainties.

## Figures and Tables

**Table 1:** Piston diameters PG-39 @ 20 °C

	*D*_p1_(0°) 35.82283 mm *D*_p2_(0°) 35.82293 mm		*D*_p1_(90°) 35.82290 mm *D*_p2_(90°) 35.82284 mm	
1st Average	35.82288 mm		35.82287 mm	
2nd Average		35.822875 mm		
Max. dev.		0.000050 mm		1.40 × 10^−6^ mm/mm
Variance *s*		0.000048 mm		1.34
Variance of mean		0.000024 mm		0.67
*k*(68.27 %) = 1.20				
k*s/*n*^1/2^		0.000029 mm		
*u*(*d*_p_)		0.000029 mm		0.80
Type A uncertainty		0.000029 mm		0.80
Type B uncertainty		0.000015 mm		0.42
*u*(*d*_p_)		0.000032 mm		0.91

**Table 2:** Cylinder diameters PG-39 @ 20 °C

	*D*_c1_(0°) 35.82433 mm *D*_c2_(0°) 35.82432 mm		*D*_c1_(90°) 35.82430 mm *D*_c2_(90°) 35.82432 mm	
1st Average	35.82433 mm		35.82431 mm	
2nd Average		35.824318 mm		
Max. dev.		0.000015 mm		0.42 × 10^−6^ mm/mm
Variance *s*		0.000013 mm		0.35
Variance of mean		0.000006 mm		0.18
*k*(68.27 %) = 1.20				
k*s/*n*^1/2^		0.000008 mm		
*u*(*d*_c_)		0.000008 mm		0.21
Type A uncertainty		0.000008 mm		0.21
Type B uncertainty		0.000015 mm		0.42
*u*(*d*_c_)		0.000017 mm		0.47

**Table 3:** Gauge effective area PG-39 @ 20 °C

	*A*_p_	*A*_c_	*A*_eff_ = (*A*_p_ + *A*_c_)/2
Area	1007.8845 mm^2^	1007.9656 mm^2^	1007.9251 mm^2^
Type A	0.001619 mm^2^	0.000425 mm^2^	0.000837 mm^2^
Type B	0.000844 mm^2^	0.000844 mm^2^	0.000844 mm^2^
*u*_tot_(*A*_e_)=			0.001189 mm^2^
*u*_tot_(*A*_e_)/*A*_e_=			1.18 × 10^−6^ mm^2^/mm^2^

**Table 4:** Fall-rate measurements

Absolute pressures		Fall-rates	Clearances	
P_0_ (kPa)	P_1_ (kPa)	d*z*/d*t* (nm/s)	*h*_Poise_ (nm)	*h*_Slip_ (nm)
95.1	193	454 ± 63	935 ± 130	849 ± 120
95.1	193	385 ± 53	884 ± 125	799 ± 110
100	241	494 ± 69	868 ± 120	794 ± 110
100	285	502 ± 70	810 ± 115	744 ± 105
100	422	665 ± 93	762 ± 110	712 ± 100

## Footnotes

1 Certain commercial equipment, instruments, or materials are identified in this paper to foster understanding. Such identification does not imply recommendation or endorsement by the National Institute of Standards and Technology, nor does it imply that the materials or equipment identified are necessarily the best available for the purpose.
